# Synthesis, Structures and Properties of Two Metal-Organic Coordination Polymers Derived from Manganese(ΙΙ), Thiabendazole and Polydentate Carboxylic Acids

**DOI:** 10.3390/molecules181214826

**Published:** 2013-12-02

**Authors:** Peng Liang, Wen-Xiu Xia, Wei-Man Tian, Xian-Hong Yin

**Affiliations:** 1 College of Chemistry and Chemical Engineering Guangxi University for Nationalities, Nanning 530006, China; E-Mails: 6628yxh@163.com (P.L.); twmhynu@163.com (W.-M.T.); 2 College of Chemistry and Chemical Engineering, Guangxi University, Nanning 530004, China; E-Mail: yxhphd@163.com

**Keywords:** thiabendazole, single-crystal structure, binuclear Mn(II) complex, hydrogen bonds

## Abstract

Two novel binuclear Mn(II) metal-organic coordination complexes [Mn_2_(TBZ)_2_(CDC)(C_2_O_4_)]_n_ (**1**), {[Mn_2_(TBZ)_2_(BDC)_0.5_(BTC)(H_2_O)_2_]·ET}_n_ (**2**), (where TBZ = thiabendazole, H_2_CDC = *trans*-1,4-cyclohexanedicarboxylic acid, H_2_C_2_O_4_ = oxalic acid, H_3_BTC = 1,3,5-benzenetricarboxylic acid, ET = ethanol, H_2_BDC = 1,4-benzenedicarboxylate) have been hydrothermally synthesized and characterized by elemental analysis, IR spectroscopy, thermogravimetric analysis, electrochemical analysis and single crystal X-ray diffraction. The X-ray structure analysis reveals that **1** is s two-dimensional layer and **2** is s one-dimensional chain. In complex **1**, it reveals 2-D layers composed of multi-(bidentate) bridging ligands (CDC and C_2_O_4_), and in **2**, the coordinated BTC ligands adopt a monodentate mode and with BDC ligands adopt alternately chelating Mn1 and Mn2 bridges into 1-D chains. The 3-D structures of the two complexes are stabilized by π-π stacking interactions and hydrogen bonds.

## 1. Introduction

During the last decades, chemists have devoted themselves to the development of new crystalline materials with a variety of properties, functions, and potential applications such as gas sorption, luminescence, molecular magnetism, nonlinear optics, catalysis, and ion-exchange [1,2,3,4,5].

To the best of our knowledge, selecting appropriate ligands is the most effective strategy for obtaining coordination polymers. New network structures utilizing both covalent and hydrogen bonds have attracted much interest recently due to their ﬂexible structural features. The auxiliary ligands containing N-donors, such as TBZ, were introduced into reaction systems so as to inhibit the expansion of polymeric frameworks to obtain the desired low dimensional coordination polymers. TBZ has aroused considerable interest in biology and medicine due to its antiproliferative activities [6,7,8,9]. It is an antimicrobial drug belonging to the benzimidazole derivative class, and has exhibited wide applications in human and veterinary medicine. The use of mixed ligands has been demonstrated to be a very effective approach for constructing diverse coordination frameworks [10,11,12,13]. However, the hybrid coordination polymers constructed by TBZ and aryl-acid combinations are rarely reported, although these two ligands are familiar to us. In this paper, we describe the syntheses of two novel binuclear Mn(II) metal-organic coordination complexes [Mn_2_(TBZ)_2_(CDC)(C_2_O_4_)]_n_ (**1**), and {[Mn_2_(TBZ)_2 _(BDC)_0.5_(BTC)(H_2_O)_2_]∙ET}_n_ (**2**), and we report the crystal structures, elemental analyses, electrochemical analysis, IR spectroscopy and thermal properties of these two novel Mn(II) complexes with TBZ ligands [14,15].

## 2. Results and Discussion

### 2.1. Crystal Structure Descriptions

The single-crystal X-ray diffraction analysis reveals that the complexes **1** and **2** crystallize in the triclinic system with space group *P-1* and *P-1*, respectively (Table 1).

**Table 1:** Crystal data and structure refinements for **1**–**2**.

	1	2
Empirical formula	C_15_H_12_MnN_3_O_4_S	C_35_H_29_Mn_2_N_6_O_11_S_2_
ormula weight	385.29	883.66
Temperature (K)	296(2) K	296(2) K
Crystal system	Triclinic	Triclinic
Space group	P-1	P-1
Unit cell dimensions (Å)		
a	9.0363(15)	10.269(2)
b	9.1349(16)	11.696(3)
c	10.1304(17)	17.101(4)
α	76.551(2)	73.414(3)
β	77.815(2)	84.529(3)
γ	74.030(2)	73.546(3)
Calculated density (Mg/m^3^)	1.657	1.555
F(000)	392	902
Volume (Å^3^), Z	772.2(2), 2	1887.6(8), 2
Absorption coefficient (mm^−1^)	1.016	0.848
θ range for data collection (°)	2.09–25.00	1.89–25.00
Data/restraints/parameters	2677/0/217	6547/585/522
Goodness-of-fit on F 2	1.055	1.039
Final Ra indices [I > 2σ(I)]	R1 = 0.0265, ωR2 = 0.0789	R1 = 0.0394, ωR2 = 0.1118
R indices (all data)	R1 = 0.0287, ωR2 = 0.0811	R1 = 0.0498, ωR2 = 0.1191

#### 2.1.1. Crystal Structures of Complex **1**

**Figure 1:**
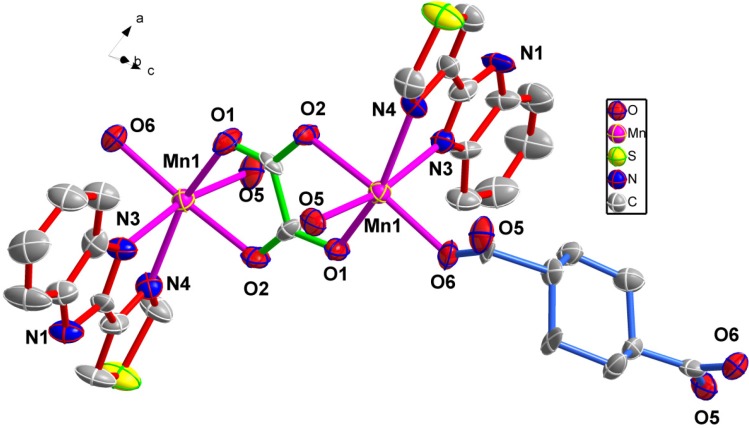
The coordination environment of **1**. All H atoms are omitted for clarity.

The self-assembly of MnCl_2_·4H_2_O with *trans*-1,4-cyclohexanedicarboxylic acid (H_2_CDC) and thiabendazole in H_2_O–CH_3_OH–DMF mixed solution was performed under weak acid conditions. If DMF was not added into the reaction system, only an unidentified ropy precipitate was obtained. If methanol was replaced by ethanol under the same conditions, we could not obtain single crystals. Single crystal X-ray diffraction analysis shows that the asymmetric unit of **1** consists of two Mn(II) ions, one completely deprotonated H_2_CDC, two thiabendazole lidangs and one [C_2_O_4_]^2−^ anion. Each metal ion is six-coordinate, the Mn1 coordinate with two nitrogen atoms from one TBZ (Mn1-N 2.2845(16)-2.3412(16) Å), four oxygen atoms from two different bridging H_2_CDC ligands and one bidentately chelating coordinating carboxylate H_2_C_2_O_4_ (Mn1-O 2.0909(14)-2.2395(14) Å), forming a distorted octahedral geometry. The environment of the two Mn atoms are similar. We can see the environment of Mn in Figure 1.

**Figure 2:**
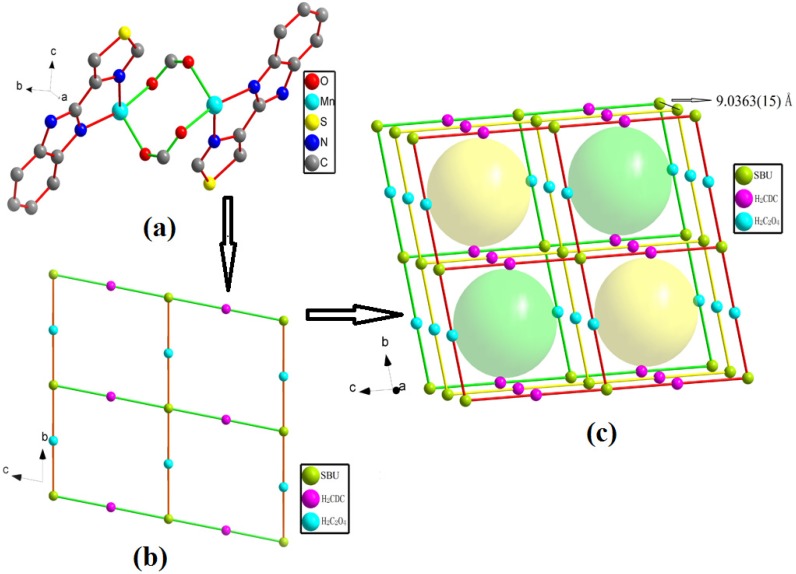
(**a**) Thermal ellipsoid-and-stick representation of SBU. (**b**) Each SBU as a four-connected node bridged by CDC and C_2_O_4_ ligands to connected with other four SBU nodes. (**c**) The spatial structure of complex **1** (green: SBU; red: H_2_CDC ligand; blue: H_2_C_2_O_4_ ligand).

**Figure 3:**
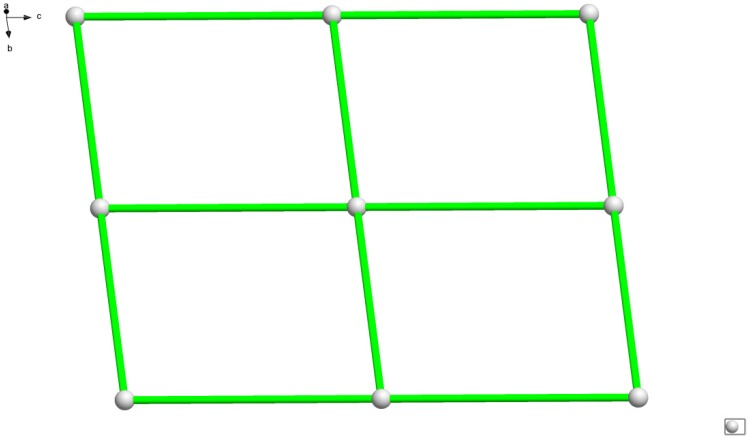
A perspective view of topological structure for complex **1** (dual-core manganese is simplified to a node).

**Figure 4:**
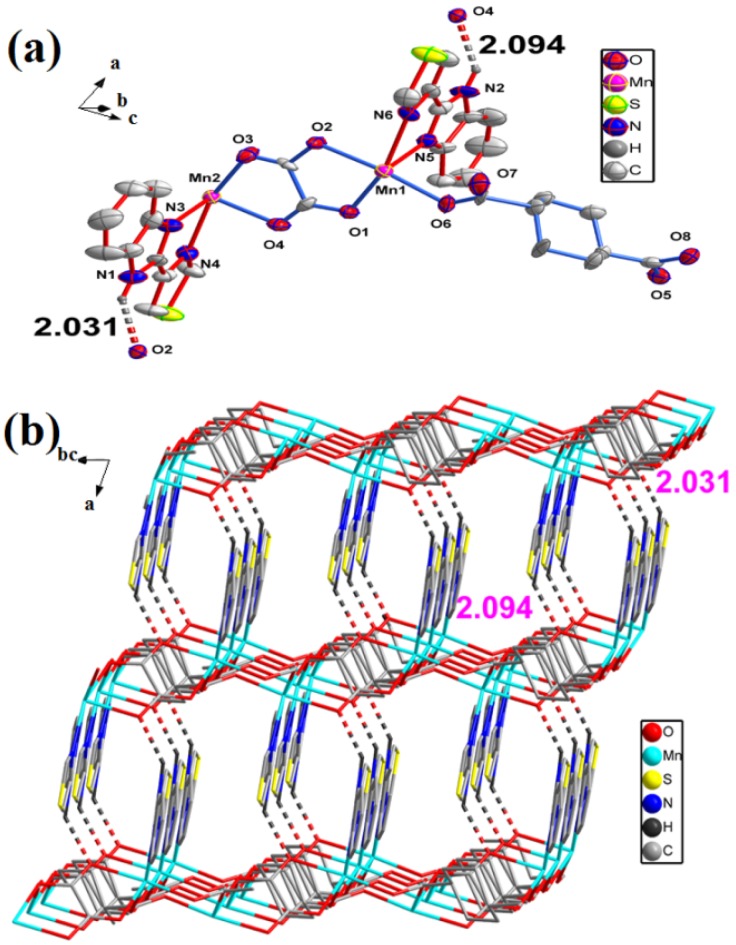
(**a**) A perspective view of H-bonding for complex **1**. Unnecessary atoms are omitted for clarity. (**b**) View of the 3-D coordination topology network created by the H-bonding and the TBZ ligand.

In **1**, the manganese bimetallic unit bridged by two carboxylic groups and two TBZ ligands, constitute a trimetallic second building unit (SBU) [Mn_2_(CO_2_)_2_(TBZ)_2_] (Figure 2a), with a non-bonding Mn1···Mn1 distance of 4.183 Å. The trimetallic SBU, as a four-connected node, is linked through bridging [C_2_O_4_]^2−^ and [CDC]^2−^ to generate an extended 2-D layer, and the distances between two layers is 9.0363(15) Å (based on the manganese atoms, Figure 2b,c). The adjacent layers are connected through N-H···O hydrogen bonds from N-H of TBZ together with a carboxylic acid O atom of [C_2_O_4_]^2−^ (N1-H1···O2), the length of them are 2.031 Å. V1 Point (Schlafli) symbol:{4^4^.6^2^} (TD10=221), Extended point symbol:[4.4.4.4.6(2).6(2)] 4-c net; uninodal net, topological type: **s**ql (Figure 3, dual-core manganese is simplified to a node). These weak interactions of hydrogen bonds play a vital role in determining the crystal packing and construction of the extended 3-D supramolecular network (Figure 4).

#### 2.1.2. Crystal Structures of Complex **2**

**Figure 5:**
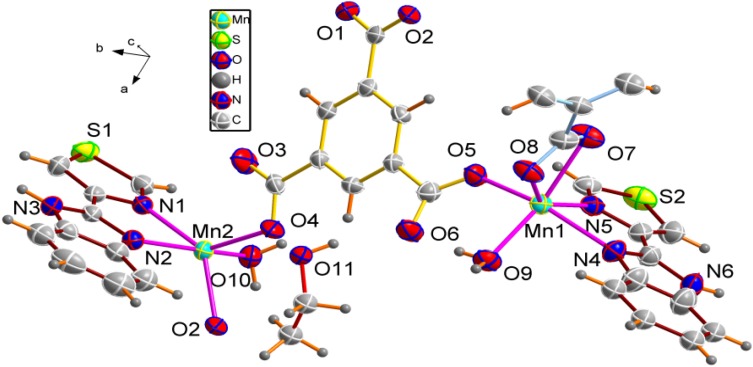
The coordination environment of **2**.

The ORTEP drawing of the fundamental building unit of **2** is shown in Figure 5. Single crystal X-ray diffraction analysis shows that the asymmetric unit of **2** consists of two Mn(II) ions, one completely deprotonated [BTC]^3−^ anion, half of a completely deprotonated [BDC]^2−^ anion, two coordinated water molecules and a free ethanol. In the structure, each Mn1 center is hexacoordinated by two nitrogen atoms (N4, N5) from one TBZ ligand, two oxygen atoms (O7, O8) from one chelating carboxylate group [BDC]^2−^, one oxygen atom (O5) from another monodentate carboxylate group [BTC]^3−^ and one oxygen atom (O9) from a coordinated water.

**Figure 6:**
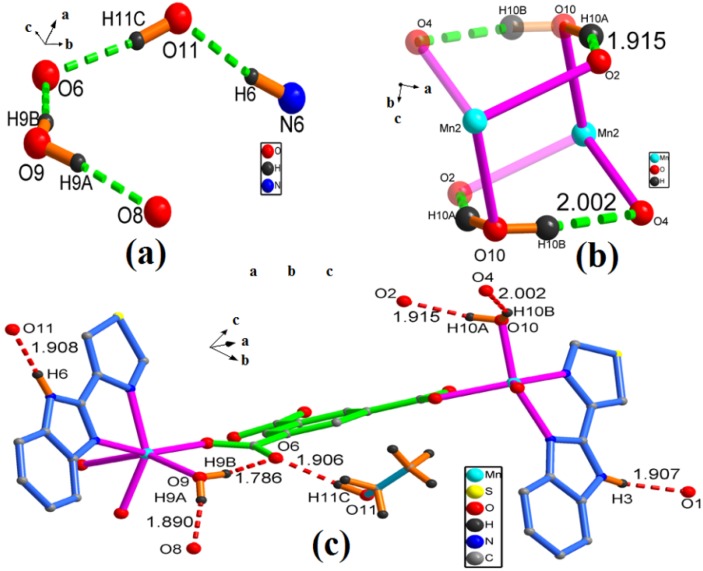
(**a**) The H-bonding chain in complex **2**, Unnecessary atoms are omitted for clarity. (**b**) View of the irregular polyhedron created by the H-bonding and the two coordinated water (O10). (**c**) A perspective view of H-bonding for complex **2**, Unnecessary atoms are omitted for clarity.

As shown in Figure 6c, there are a wealth of hydrogen bonds in **2**. The adjacent chains are connected through N-H···O and O-H···O hydrogen bonds from the N-H of TBZ and O-H of ethanol. The hydrogen bonds from N-H of TBZ, together with a carboxylic O atom of BTC and hydroxyl O atom of ethanol (N3-H3···O1, N6-H6···O11) shows lengths of 1.907 and 1.908 Å, respectively.

**Figure 7:**
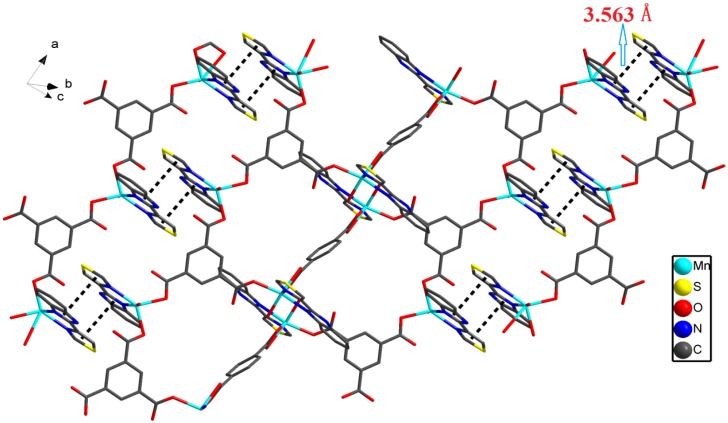
The face to face π–π stacking interactions between two heterocyclic of different TBZ ligands in complex **2**, all H atoms are omitted for clarity.

At the same time, the hydrogen bonds from O-H of ethanol together with carboxylic O atom of another chain. Four additional hydrogen bonds are O-H···O (the length of O9-H9A···O8 is 1.890 Å, O9-H9B···O6 is 1.785 Å, O10-H10A···O2 is 2.002 Å and O10-H10B···O4 is 1.914 Å) within the chain, the oxygen atoms of coordinated water provide hydrogens (Figure 6a). Two coordinated water molecules in adjacent Mn2 ions are hydrogen-bonded to each other, these hydrogen bonds with other atoms form an irregular polyhedron (Figure 6b). As complex **2** is a 1-D chain, and the TBZ ligands are arranged alternately on both side of the chain, there will be a rich π–π stacking interaction between these chains. This will produce a kind of π–π stacking interactions, whose distances is 3.563 Å (Figure 7).

**Figure 8:**
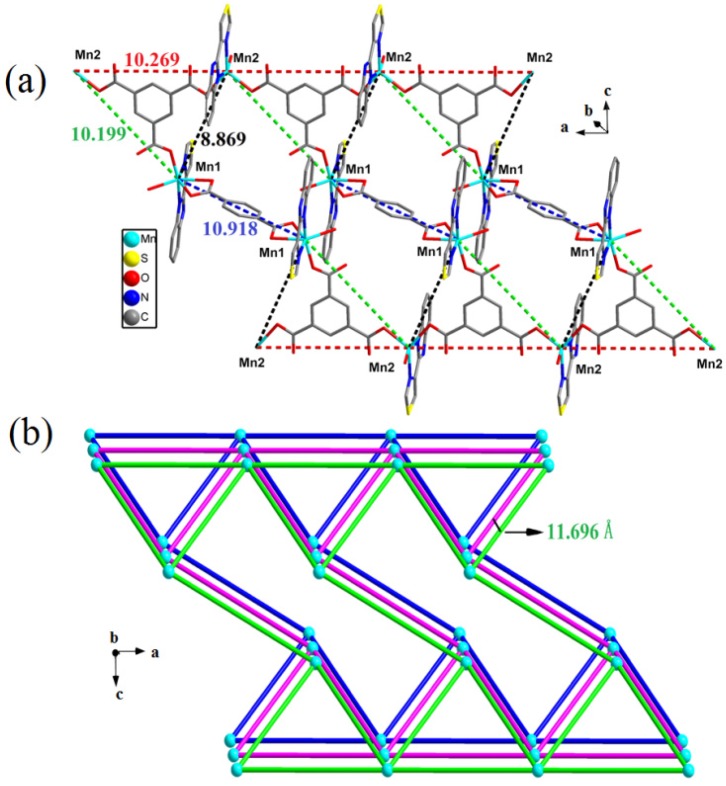
(**a**) Perspective view of 2-D network. (**b**) Schematic illustration of offset stacking of adjacent three 2-D single layers in compound **2**. (Color code: blue, Mn).

Each Mn2 center is five-coordinated by two nitrogen atoms (N1, N2) from one TBZ ligand, two oxygen atoms (O2, O4) from two different monodentate carboxylate groups [BTC]^3−^ and one oxygen atom (O10) from a coordinated water. In **2**, the Mn-O bonds lengths vary from 2.128(2) to 2.293(2) Å, while the Mn–N bonds lengths vary from 2.198(3) to 2.250(3) Å. Each BTC ligand connects three Mn(II) centers leading to a triangle(Mn1, Mn2, Mn2), and the three sides of the triangle lengths measure 10.199, 8.869 and 10.269 Å, respectively. Two Mn1 metal centers are bridged by a completely deprotonated [BDC]^2−^ anion, the distance between them is 10.918 Å, while the Mn1 centers connect with another triangle (Mn1, Mn2, Mn2) (Figure 8a).

**Figure 9:**
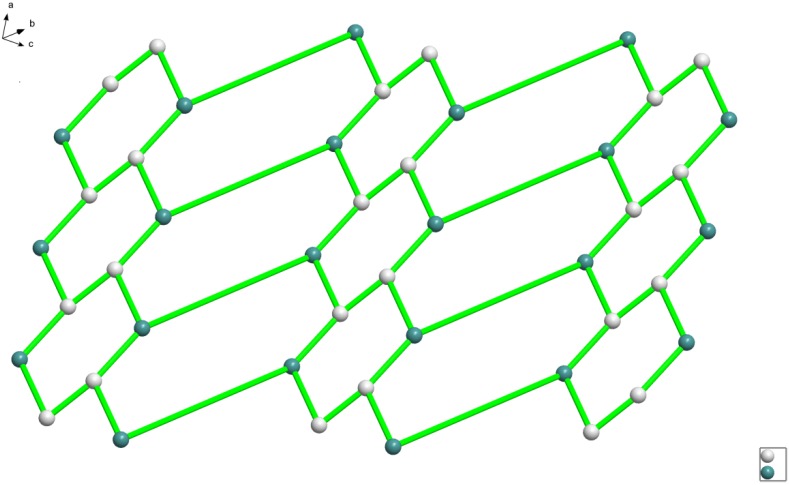
A perspective view of the topological structure of complex **2**.

**Figure 10:**
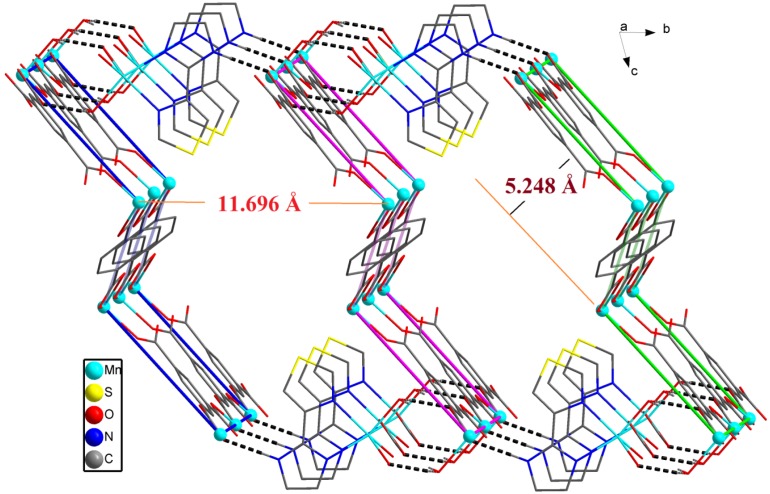
The three-dimensional architecture of the crystal via H-bonding (hydrogen bonds: yellow). Unnecessary atoms are omitted for clarity.

Topology symbol {6^3^} (TD10=166); extended point symbol: [6.6.6]; 3,3-c net; uninodal net; topological type: hcb (Figure 9). The H_2_BDC ligand connects two triangle metal frames, and the distance within the planes of two metal frames is 5.248 Å. It is noteworthy that the adjacent Mn2 metal centers lead to a straight chain and the distance between Mn2-Mn2 is 10.269 Å, and the distance between two chains is 11.696 Å (Figure 8b). The π–π stacking interactions make the 1-D chains to generate 2-D network, and the hydrogen bonds interlink make the 2-D layers to generate 3-D supramolecular architectures (Figure 10).

### 2.2. Thermal Analysis

**Figure 11:**
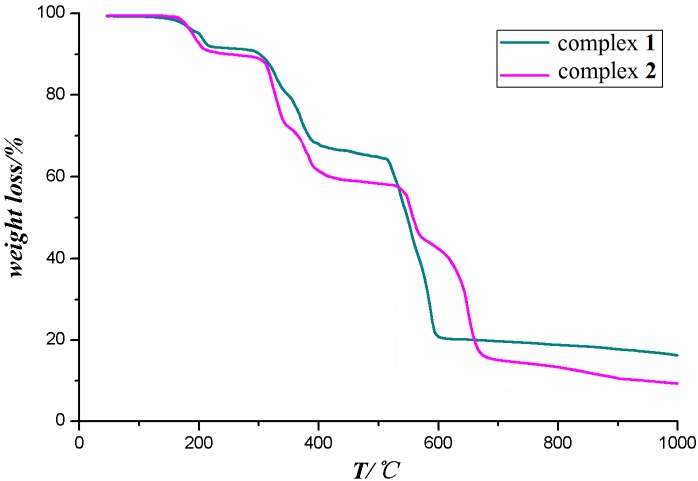
The TG curves of the two complexes.

Thermogravimetric analyses show that compounds **1** and **2** have a low thermal stability as illustrated in Figure 11. Complex **1** displays mainly three weight loss steps. The first starts from 172 to 228 °C with a mass loss of 10.56%, corresponding to loss of a [C_2_O_4_]^2−^ group (calcd. 11.37%), then it is stable up to 286 °C. The second stage occurs in the 286–395 °C range with a mass loss of 21.72% (calcd. 22.15%) that correlates with elimination of [CDC]^2−^. The third stage in the 516–594 °C range corresponds to release of TBZ molecules with a weight loss of 50.73% (calcd. 51.91%). There is no obvious weight loss before 158 °C in the TG curve of compound **2**. Beyond this temperature, two indiscernible processes with a total weight loss of 10.15% corresponding to the combustion of water molecules (calcd. 5.36%) and free ethanol (calcd. 4.24%) was observed. Beyond 308 °C, two indiscernible processes with a total weight loss of 32.48% corresponding to the combustion of [BTC]^3−^ (calcd. 23.78%), [BDC]^2−^ (calcd. 9.42%) was observed. When the temperature continues rising, the product lost 44.72% of the total weight beyond 526 °C, which is related to the loss of two TBZs (calcd. 45.33%).

### 2.3. Electrochemical Properties

**Figure 12:**
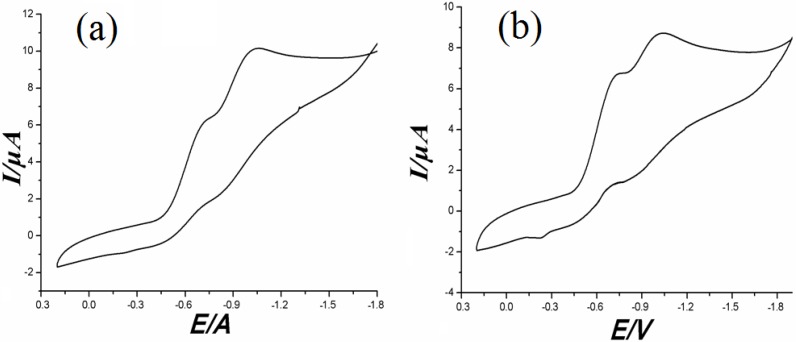
(**a**) Cyclic voltammograms of complex **1** measured at room temperature. (**b**) Cyclic voltammograms of complex **2** measured at room temperature.

The electrochemical behavior of complex **1**–**2** in LiClO_4_ (0.05 mol·L^−1^) and ethanol solution has been investigated by cyclic voltammetry in the potential range from −1.8 to 0.3 V. The resulting cyclic voltammogram (CV) of complex **1** is shown in Figure 12a.

Only two reduction peaks (E_pc_ = −1.034 V, E_pc_ = −0.712 V), and no significant oxidation peak is seen in complex **1**, which shows that Mn^II^ can easily be restored continuously, and is not easily oxidized. Complex **2** displays two pairs of quasi-reversible oxidation and reduction waves with a reduction potential ranging from −0.66 to −0.77 V, −0.97 to −1.18 V and an oxidation potential ranging from −0.17 to −0.26 V, −0.79 to −0.94 V (Figure 12b). The reduction and the oxidation peaks were assigned to the Mn^III^/Mn^II^ and Mn^II^/Mn^I^ couples which correspond to two electron-transfer processes.

## 3. Experimental

### 3.1. Materials and Instrumentation

All chemicals were commercial materials of analytical grade and used without purification. Elemental analysis for C, H, and N was carried out on a Perkin-Elmer 2400 II elemental analyzer (Waltham, MA, USA). The FT-IR spectrum was obtained on a PE Spectrum One FT-IR Spectrometer Fourier transform infrared spectrometer in the 4000–400 cm^−1^ region, using KBr pellets. Powder X-ray diffraction patterns were obtained using a pinhole camera (Bruker, Munich, Germany) operating with a point focused Ni-filtered Mo-*Kα* radiation in the 2θ range from 5 to 50° with a scan rate of 0.08° per second. Cyclic voltammetry were performed on a CHI 660C electrochemical workstation. Thermogravimetric analysis was performed on a Perkin-Elmer TG/DTA 6300 thermal analyzer under a N_2_ atmosphere at a heating rate of 10 °C·min^−1^.

### 3.2. Synthesis of Complexes **1**–**2**

#### 3.2.1. Synthesis of [Mn_2_(TBZ)_2_(CDC)(C_2_O_4_)]_n_ (**1**)

A solution of H_2_CDC (84.1 mg, 0.5 mmol) in DMF (3 mL) was added dropwise with stirring at room temperature to a solution of TBZ (100.3 mg, 0.5 mmol) and MnCl_2_·4H_2_O (97.9 mg, 0.5 mmol) in the mixture of water (10 mL) and methanol (5 mL). Then an aqueous solution of oxalic acid was added dropwise with stirring to adjust the pH value of the solution to 5. The resulting mixture was sealed in a 25 mL Teflon-lined stainless reactor, and kept under autogenous pressure at 130 °C for 72 h, and then slowly cooled to room temperature at a rate of 5 °C per hour. Yellow block-shaped crystals suitable for X-ray diffraction were isolated directly, washed with carbinol and dried at room temperature (yield: 52%, based on Mn). Anal. Calcd. for C_15_H_12_MnN_3_O_4_S (%): C, 46.76; H, 3.14; N, 10.91; Found: C, 46.58; H, 3.17; N, 10.86. IR data (cm^−1^): 3104 (s), 1673 (vs), 1609 (vs), 1434 (s), 1317 (s), 1259 (m), 797 (m), 743 (m).

#### 3.2.2. Synthesis of {[Mn_2_(TBZ)_2_(BDC)_0.5_(BTC)(H_2_O)_2_]·ET}_n_ (**2**)

TBZ (100.2 mg, 0.5 mmol) and H_3_BTC (105.6 mg, 0.5 mmol) were dissolved in the mixture of ethanol (6 mL) and DMF (5 mL). Then an aqueous solution of oxalic acid was added dropwise with stirring to adjust the pH value of the solution to 6. At last, aqueous solution of MnCl_2_·4H_2_O (98.3 mg, 0.5 mmol) and H_2_BDC (83.1 mg, 0.5 mmol) (10 mL) was added. The resulting mixture was sealed in a 25 mL Teﬂon-lined stainless reactor, kept under autogenous pressure at 130 °C for 72 h, and then slowly cooled to room temperature at a rate of 5 °C per hour. Yellow block-shaped crystals suitable for X-ray diffraction were isolated directly, washed with carbinol and dried at room temperature (yield: 44%, based on Mn). Anal. Calcd. for C_35_H_29_Mn_2_N_6_O_11_S_2_ (%): C, 47.57; H, 3.31; N, 9.51. Found: C, 47.44; H, 3.26; N, 9.56. IR data (cm^−1^): 3318 (vs), 3104 (s), 1617 (vs), 1557 (vs), 1434 (m), 1372 (s), 1323 (m), 1232 (s), 1010 (m), 852 (w), 739 (s).

### 3.3. X-ray Structure Determination

Diffraction experiments for **1**–**2** were carried out with Mo-*Kα* radiation using a BRUKER SMART APEX CCD diffractometer at 296K. The structures were solved by direct methods and refined with full-matrix least-squares on *F^2^* using SHELXS-97 and SHELXL-97 [16,17]. All non-hydrogen atoms were refined anisotropically. Hydrogen atoms were placed at geometrically calculated positions by using a riding model. A summary of the crystallographic data and structure refinements was shown in Table 1, selected bond lengths and angles of the complexes were listed in Table 2, and hydrogen bond geometries were given in Table 3. Crystallographic data for the structures reported here have been deposited with CCDC (Deposition No. CCDC-953643 (**1**), No. CCDC-956333 (**2**). These data can be obtained free of charge via http://www.ccdc.cam.ac.uk/conts/retrieving.html or from CCDC, 12 Union Road, Cambridge CB2 1EZ, UK, E-mail: deposit@ccdc.cam.ac.uk.

**Table 2:** Selected bond lengths (Å) and angles (°) for **1**–**2**.

**Complex 1**			
Mn(1)-O(6)	2.0909(14)	Mn(1)-O(1)	2.1676(13)
Mn(1)-O(5)	2.1408(14)	Mn(1)-O(2)	2.2395(14)
Mn(1)-N(3)	2.2845(16)	Mn(1)-N(4)	2.3412(16)
O(6)-Mn(1)-O(5)	101.47(6)	O(6)-Mn(1)-O(1)	92.49(5)
O(5)-Mn(1)-O(1)	86.84(6)	O(6)-Mn(1)-O(2)	165.71(5)
O(5)-Mn(1)-O(2)	85.86(6)	O(1)-Mn(1)-O(2)	75.53(5)
O(6)-Mn(1)-N(3)	89.00(6)	O(5)-Mn(1)-N(3)	168.50(6)
O(1)-Mn(1)-N(3)	97.54(6)	O(2)-Mn(1)-N(3)	84.97(6)
O(6)-Mn(1)-N(4)	103.07(6)	O(5)-Mn(1)-N(4)	99.99(6)
O(1)-Mn(1)-N(4)	161.27(6)	O(2)-Mn(1)-N(4)	87.50(5)
N(3)-Mn(1)-N(4)	72.70(6)		
**Complex 2**			
Mn(1)-O(9)	2.128(2)	Mn(1)-O(5)	2.141(2)
Mn(1)-O(8)	2.214(2)	Mn(1)-N(4)	2.250(3)
Mn(1)-N(5)	2.260(3)	Mn(1)-O(7)	2.293(2)
Mn(2)-O(4)	2.148(2	Mn(2)-O(10)	2.168(3)
Mn(2)-N(2)	2.198(3)	Mn(2)-O(2) #1	2.234(2)
Mn(2)-N(1)	2.267(2)	Mn(2)-O(1) #1	2.546(2)
O(9)-Mn(1)-O(5)	88.13(9)	O(9)-Mn(1)-O(8)	89.50(9)
O(5)-Mn(1)-O(8)	156.11(9)	O(9)-Mn(1)-N(4)	89.61(10)
O(5)-Mn(1)-N(4)	95.15(9)	O(8)-Mn(1)-N(4)	99.97(9)
O(9)-Mn(1)-N(5)	122.53(9)	O(5)-Mn(1)-N(5)	87.21(9)
O(8)-Mn(1)-N(5)	146.80(9)	N(4)-Mn(1)-N(5)	74.05(10)
O(9)-Mn(1)-O(7)	147.37(9)	O(5)-Mn(1)-O(7)	101.33(9)
O(8)-Mn(1)-O(7)	57.98(8)	N(4)-Mn(1)-O(7)	93.30(10)
N(5)-Mn(1)-O(7)	89.36(8)	O(4)-Mn(2)-O(10)	95.77(10)
O(4)-Mn(2)-N(2)	102.42(9)	O(10)-Mn(2)-N(2)	159.82(9)
O(4)-Mn(2)-O(2) #1	90.23(8)	O(10)-Mn(2)-O(2)	94.88(10)
N(2)-Mn(2)-O(2) #1	93.71(9)	O(4)-Mn(2)-N(1)	129.59(8)
O(10)-Mn(2)-N(1)	87.23(9)	N(2)-Mn(2)-N(1)	74.50(9)
O(2)-Mn(2)-N(1) #1	139.79(8)	O(4)-Mn(2)-O(1) #1	143.36(8)
O(10)-Mn(2)-O(1) #1	81.38(10)	N(2)-Mn(2)-O(1) #1	89.02(8)
O(2)-Mn(2)-O(1) #1	54.01(7)	N(1)-Mn(2)-O(1) #1	86.92(8)

Symmetry codes: #1 x+1, y, z.

**Table 3:** Hydrogen-bond geometry (Å) for complexes.

D-H···A	D-H	H···A	D···A	D-H···A	Symmetry codes
Complex **1**					
N1-H1···O2	0.860	2.031	2.807	146.17	x−1, y, z
Complex **2**					
O11-H11C···O6	0.850	1.906	2.744	168.36	
N3-H3···O1	0.860	1.907	2.701	152.85	−x, −y+1, −z+1
N6-H6···O11	0.860	1.908	2.752	166.86	x, y−1, z
O9-H9B···O6	0.850	1.785	2.626	169.96	
O9-H9A···O8	0.850	1.890	2.731	170.01	−x, −y, −z
O10-H10B···O4	0.850	2.002	2.784	152.57	−x+1, −y, −z+1
O10-H10A···O2	0.850	1.914	2.696	152.22	−x, −y, −z+1

## 4. Conclusions

In summary, two novel binuclear Mn(II) coordination complexxes were obtained with the aid of thiabendazole and an aromatic carboxylic acid. X-ray structure analysis reveals that complex **1** forms a two-dimensional layer and **2** is a one-dimensional chain. Hydrogen bonds interlinking and π–π stacking interactions make them to generate 3D supramolecular architectures. In addition, electrochemical measurements reveal that the two complexes exhibit good redox potential at room temperature. The thermal decomposition process and powder X-ray diffraction of the complexes were also investigated.
